# Antimicrobial Surfaces: Stainless Steel Functionalized with the Essential Oil Component Vanillin

**DOI:** 10.3390/ijms252212146

**Published:** 2024-11-12

**Authors:** Serena Medaglia, Ángela Morellá-Aucejo, María Ruiz-Rico, Félix Sancenón, Luis A. Villaescusa, Ramón Martínez-Máñez, M. Dolores Marcos, Andrea Bernardos

**Affiliations:** 1 Instituto Interuniversitario de Investigación de Reconocimiento Molecular y Desarrollo Tecnológico (IDM), Universitat Politècnica de Valencia, Universitat de València, Camino de Vera s/n, 46022 Valencia, Spain; sermed@idm.upv.es (S.M.); ngemoau@upvnet.upv.es (Á.M.-A.); fsanceno@upvnet.upv.es (F.S.); lvillaes@qim.upv.es (L.A.V.); rmaez@qim.upv.es (R.M.-M.); 2 Centro de Investigación Biomédica en Red de Bioingeniería, Biomateriales y Nanomedicina, Instituto de Salud Carlos III, 28029 Madrid, Spain; 3 Unidad Mixta UPV-CIPF de Investigación en Mecanismos de Enfermedades y Nanomedicina, Universitat Politècnica de València, Centro de Investigación Príncipe Felipe, C/Eduardo Primo Yúfera 3, 46100 Valencia, Spain; 4 Instituto Universitario de Ingeniería de Alimentos (FoodUPV), Universitat Politècnica de València, Camino de Vera s/n, 46022 Valencia, Spain; mruizrico@ucc.ie; 5 Departamento de Química, Universidad Politécnica de Valencia, Camino de Vera s/n, 46022 Valencia, Spain; 6 Unidad Mixta de Investigación en Nanomedicina y Sensores, Universitat Politècnica de València, Instituto de Investigación Sanitaria La Fe, Av Fernando Abril Martorell 106, 46026 Valencia, Spain

**Keywords:** stainless steel, antimicrobial surface, vanillin, silica, biofilm

## Abstract

Pathogenic microorganisms can adhere to solid surfaces, leading to the formation of biofilms, thus building a physical barrier hindering the penetration and diffusion of antimicrobial compounds. In this context, the use of natural antimicrobial compounds, such as essential oil components, as substitutes for common synthetic antimicrobials in the fight to prevent antimicrobial resistance is explored. As stainless steel is one of the most widely used surfaces in different industries, we have developed an innovative antimicrobial treatment for stainless steel surfaces based on a multi-step functionalization process, in which the stainless steel surface is coated with a silica layer to which a vanillin derivative is covalently attached. The surface was analyzed by microscopy studies, indicating the correct immobilization on the surfaces. Antimicrobial studies (viability and bacterial adhesion assays) were performed against the bacteria *Staphylococcus epidermidis*, which is one of the most frequent causes of nosocomial infections. The results of the microbiological studies showed that vanillin-functionalized stainless steel surfaces reduce the bacteria viability by 100% and the biofilm formation on the stainless steel surface by 75% compared with non-functionalized surfaces, highlighting the contact-killing and adhesion resistance properties of the developed surface. Additional cycles using the functionalized surfaces showed good maintenance of the antimicrobial coating efficacy. Moreover, the surfaces coated with an intermediate silica layer demonstrated much greater antimicrobial activity than surfaces in which the active molecule was directly functionalized on the stainless steel surface.

## 1. Introduction

Nosocomial infections are one of the most critical problems occurring in the healthcare system, becoming one of the leading causes of mortality and increased morbidity in hospitalized patients [1]. One of the main reasons for these infections is the microbial ability to adhere to solid surfaces (biotic or abiotic). This ability causes the formation of polymeric structures called biofilms [2]. The biofilms act as a trap for nutrients and a physical barrier hindering the penetration and diffusion of antibiotics and common disinfectants [2,3,4]. Biofilm formation begins with the adhesion of single planktonic cells to a surface. These adherent cells grow into microcolonies that then proliferate further into mature biofilms. Mature biofilms produce extracellular matrix (ECM) components critical for maintaining cellular aggregates’ distinctive structures [5,6].

*Staphylococcus epidermidis* is a *Staphylococcus* species that is part of the normal microbiota of the mucosa and skin of humans and other mammals [7]. For this reason, *S. epidermidis* has long been considered an avirulent species. However, nowadays, this bacterium is regarded as one of the most frequent causes of nosocomial infections, particularly those related to medical devices [8], being one of the most important etiological agents of device-associated infections. For example, *S. epidermidis* may be involved in the infections of implants, vascular grafts, cardiac devices, etc. The ability of *S. epidermidis* to form biofilms is a key virulence factor that enables the bacteria to adhere to both biotic and abiotic surfaces, and to resist the host immune system and antibiotics [8,9]. This ability is one of the most critical resistance mechanisms developed by this microorganism [7].

An effective strategy to prevent biofilm formation in biotic and abiotic surfaces is to deposit an antimicrobial layer on the material’s surface. Several approaches have been employed in the search for effective antimicrobial surfaces [2,10,11], including adhesion resistance, contact killing, and biocide leaching [12,13,14]. Among these approaches, contact killing seems to be the most promising route; this approach involves chemical or physical modifications to bind an antimicrobial agent to the surface [12,15,16]. Some of the most commonly applied strategies for surface modification are adsorption (via electrostatic interactions), ligand–receptor coupling, and covalent attachment [17]. Covalent binding offers several advantages by providing the most stable bonds between the bioactive compound and the surface [17]. Some studies have reported the immobilization on different surfaces (plastic, titanium, stainless steel, and others) of specific bioactive molecules such as antibacterial peptides, including magainin [17], nisin [16,17], and lysozyme [18], or the immobilization of biopolymers such as chitosan [17,19,20]. However, depending on the application, medical or food, they have limited efficiency or toxicity, and their use is associated with side effects such as hypersensitivity and inflammatory responses [2]. Moreover, the indiscriminate use of classical antimicrobials has generated a more significant problem: antimicrobial resistance [2]. Antimicrobial resistance (AMR) occurs when bacteria, viruses, fungi, or parasites change over time and no longer respond to medicines, making infections more challenging to treat and increasing the risk of disease spread, severe illness, and death. As a result of drug resistance, antibiotics and other antimicrobial medicines become ineffective, and the treatment of infections becomes increasingly difficult or impossible [21].

In this context, the design of antimicrobials avoiding synthetic antibiotics is of great importance, and numerous studies have focused on the potential use of natural compounds [22,23,24]. General attention has been directed toward studying the properties of essential oils (EOs) as a possible alternative to synthetic antimicrobial agents [25]. EOs are aromatic oily liquids found in all plant organs (flowers, buds, seeds, leaves, twigs, bark, herbs, wood, fruits, and roots). EOs contain a wide range of secondary metabolites with antioxidant, antiseptic, antimicrobial, anti-inflammatory, and anesthetic properties [26]. The antimicrobial activity and antibiofilm activity of some essential oil components (EOCs) (such as carvacrol, eugenol, thymol, and vanillin) have been described against certain pathogenic and spoilage microorganisms [27,28,29,30]. The antimicrobial activity of EOCs is not ascribed to a single mechanism but to a cascade of reactions (cell wall degradation, cytoplasmic membrane damage, membrane protein damage, coagulation of the cytoplasm, and proton power depletion) [31] involved in the destruction of bacterial cells [32]. Moreover, EOCs have also been reported to penetrate biofilms and kill protected bacteria [27].

**Figure 1:**
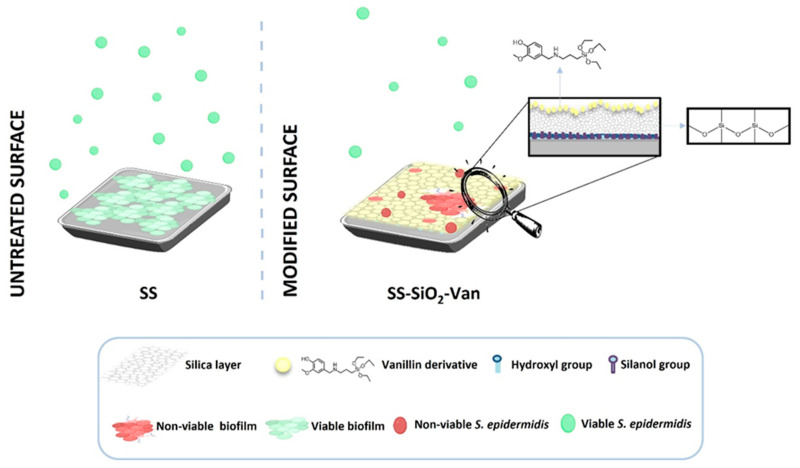
Antimicrobial surfaces based on the bioactive molecule vanillin functionalized on stainless steel surfaces.

Based on the above, we report herein the functionalization of AISI 316L stainless steel (SS), which is a material with a remarkable number of applications in industrial and medical fields, with the bioactive antimicrobial molecule vanillin to develop a contact-killing antimicrobial surface [33,34,35]. The covalent anchoring of vanillin inhibits its rapid elimination (vanillin is highly volatile) and enhances its antimicrobial activity, according to our previous studies [36,37]. We additionally focused our study on *S. epidermidis*, one of the most frequent bacteria responsible for chronic biofilm-associated infections in tissues, such as the skin and the respiratory tract, and in the formation of biofilms in medical devices (see Figure 1) [8,9].

## 2. Results and Discussion

### 2.1. Design, Preparation, and Characterization of SS-SiO_2_-Van Surface

Following an increasing consumer interest in natural products, our research has been inspired by the use of antimicrobial essential oil components (EOCs) to replace synthetic products. In fact, previous research has demonstrated the use of EOCs as antimicrobials, but few examples have studied their use in surface coatings. For example, Nielsen et al. [38] coated stainless steel and polyethylene surfaces with isoeugenol by physical adsorption to inhibit *Staphylococcus aureus*, *Listeria monocytogenes*, and *Pseudomonas fluorescens*. Guarda et al. [39] coated polypropylene films with microcapsules containing thymol and carvacrol. These coatings showed antimicrobial activity against *Staphylococcus aureus*, *Listeria innocua*, and *Escherichia coli*. Other studies [40,41,42,43,44] have also demonstrated the antimicrobial activity of surfaces coated with EOCs. Table 1 summarizes the functionalization of surfaces with EOCs for antimicrobial applications.

In relation to vanillin, its use as a natural antimicrobial compound has already been studied [36,37]. The antimicrobial activity of Van has been attributed to the interaction of the Van phenolic group with microbial cell membranes, which causes the leakage of ions and cytoplasmic content and can, therefore, lead to cellular disintegration. This translates into a bactericidal effect on microorganisms [32,45,46], even if it is noted, according to some studies, that, in some situations, Van has only bacteriostatic activity, producing an inhibition of the microorganism without killing it. In fact, in the study by Cava-Roda et al. (2010) [46], it was observed that the effect of vanillin in milk was bacteriostatic rather than bactericidal. The cause of this effect is the formation of hydrogen bonds and hydrophobic interactions between proteins and vanillin [47]. Despite these studies on the activity of the Van molecule, to the best of our knowledge, Van has not previously been used as an antibacterial function for SS surface coating.

**Figure 2:**
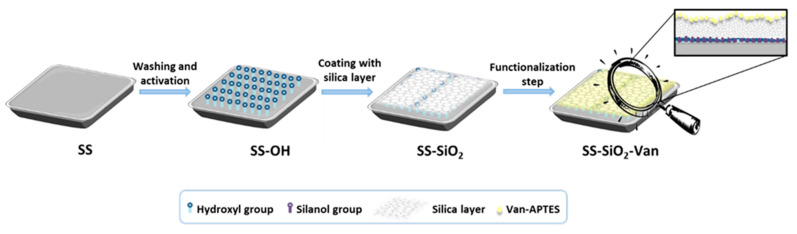
Schematic illustration of the SS surface functionalization.

During our research, we first evaluated the possibility of the direct bonding between the steel and the bioactive molecule (vanillin), but several problems were found (corresponding comment in Section 2.2), such as the leaching of the active sites, a non-antimicrobial activity, and a low reproducibility in the coating of materials. Because of that, we followed a different procedure, in which we first coated the SS surface with an amorphous silica layer to which a vanillin derivative was covalently attached. In particular, the surface of the 316L stainless steel was functionalized following a three-step procedure (Figure 2): (i) pretreatment and activation of the stainless steel surface, (ii) formation of a silica layer, and (iii) immobilization of a vanillin derivative. Standard techniques were used for the characterization of all of the synthesized materials.

**Figure 3:**
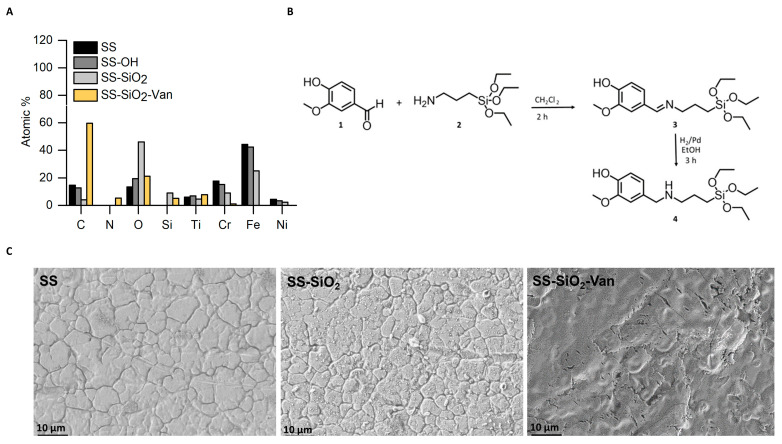
(**A**) HR-FESEM EDX analysis of the untreated and modified surfaces. (**B**) Synthetic protocol of the **4** (Van-APTES) derivative. (**C**) HR-FESEM images of untreated and modified surface: stainless steel surface (SS), stainless steel surface with the silica layer (SS-SiO_2_), and stainless steel surface with the silica layer–vanillin derivate (SS-SiO_2_-Van).

The first step in the SS functionalization was the preparation of the stainless steel surface by ultrasonication in acetone, ethanol, and, finally, in water. After the washing procedure, the SS surface was treated with a piranha solution to induce the formation of hydroxyl groups on the metal surface [48,49]. The piranha solution was selected because it reduces surface contamination and oxidizes and hydroxylates the surface of most of the metals, as it can react with the outer oxide layer of metals and expose reactive hydroxyl groups [48]. These hydroxyl groups are formed by the dissociative chemisorption of water molecules, and it is generally considered that hydration and hydroxylation occur at exposed lattice metal ion sites on the surface [50]. These hydroxyl groups are essential for the subsequent coating step of the materials, as they are essential in the formation of strong covalent bonds with the silanol groups of the coating silica [51,52]. This activation is critical because the amount of hydroxyl groups obtained during the activation process strongly influences the initiation of silane attachment and, hence, the formation of the silica film [48]. In our case, SS surfaces were exposed to the piranha solution at a reduced time (10 min) to avoid the corrosion of the SS surface [49,53,54]. Thus, the SS and the obtained SS-OH surfaces differ only in the presence of generated hydroxyl groups [43]. The SS and SS-OH materials were analyzed with the EDX detector of the HR-FESEM. As can be seen from the data obtained (Figure 3A), there is an increase in the percentage of O on the SS-OH surface, thus confirming a correct activation of the material.

In the second functionalization step, the SS-OH surface was treated with tetraethyl ortho silicate (TEOS) using a sol-gel procedure. The hydrolysis of the TEOS produces silanol groups (Si-OH) in the presence of water [48], which facilitates quick adsorption on the SS-OH surface to finally form metal–siloxane (M-O-Si-OH) covalent bonds. Moreover, the simultaneous condensation of silanol groups with silicate entities already attached to the metal surface contributes to the formation of a Si-O-Si network that covers the metal surface, giving rise to the SS-SiO_2_ material [32]. The surface of the SS-SiO_2_ was also analyzed with HR-FESEM-EDX to confirm the expected coating. The data obtained (Figure 3A) showed that the surfaces of the SS-OH and SS-SiO_2_ materials differ in the additional presence of the Si element in SS-SiO_2_ and in the decrease in the percentage of Fe, from 42.32% in SS-OH to 25.1% in SS-SiO_2_. This indicates the successful deposition of an amorphous silica coating, which ensures the presence on the surface of the necessary silanol groups for further functionalization. Thus, the silanol groups facilitate the covalent attachment of organic molecules onto the surface, resulting in stable functionalization due to their excellent thermal, mechanical, and chemical stability [55].

Finally, the last step of the functionalization process requires anchoring the antimicrobial entity to the surface of the SS-SiO_2_ material. For that purpose, a trialkoxysilane derivative containing the EOC vanillin (**4**) was prepared. Derivative **4** was synthesized from the reaction of vanillin aldehyde (**1**) with (3-aminopropyl) triethoxysilane (APTES) (**2**), resulting in the formation of the imine derivative **3** (Figure 3B). In a second step, the imine derivative **3** was reduced by chemical hydrogenation using H_2_ and palladium on carbon (Pd/C) as a catalyst to give the amine derivative **4** (also named Van-APTES) (Figure 3B).

The ^1^H NMR, ^13^C NMR, ^29^Si NMR, and HRMS data (Section 3.2) confirmed the correct synthesis of the derivative **4**. After the first step of the synthesis, the proton signal at 8.12 ppm confirmed the imine bond formation between vanillin and APTES to give derivative **3**. In addition, the proton signals at 3.74–3.68 ppm and 1.11 ppm confirmed the presence of the ethoxy groups. In the second step, the ^1^H NMR data confirmed the reduction of the imine bond, as the proton signal of the imine group (8.12 ppm) disappeared, and a new proton signal was observed at 2.45 ppm. Additionally, proton signals at 3.75–3.72 ppm and 1.17–1.10 ppm confirmed the presence of the ethoxy silane groups, demonstrating that the ethoxy groups in the final compound **4** were not hydrolyzed during the reaction.

For the final functionalization, the SS-SiO_2_ surface was in contact with the vanillin derivative (**4**) to obtain the SS-SiO_2_-Van material. Covalent binding to the surface occurs by hydrolysis of the ethoxy groups in **4** and condensation with the silanols of the SS-SiO_2_ surface, leading to the formation of covalent Si-O-Si bonds [56,57]. The analysis obtained with HR-FESEM-EDX (Figure 3C) of the SS-SiO_2_ and SS-SiO_2_-Van surfaces shows the presence of nitrogen, the decrease in the percentage of Fe (25.1 % for SS-SiO_2_ and 0% for SS-SiO_2_-Van) and the increase in the percentage of C (59.7% for SS-SiO_2_-Van and 4.1% for SS-SiO_2_), confirming the immobilization of the vanillin derivative **4** on the surface.

With regard to the morphology of the materials, the images obtained by HR-FESEM (Figure 3C) confirmed that the activation and immobilization process did not change the integrity of the stainless steel (SS) (Figure 3C) in the modified materials (SS-SiO_2_ and SiO_2_-Van) (Figure 3C). The figures also show a good homogeneity of the coatings, both in SS-SiO_2_ and SS-SiO_2_-Van (Figure 3C).

**Figure 4:**
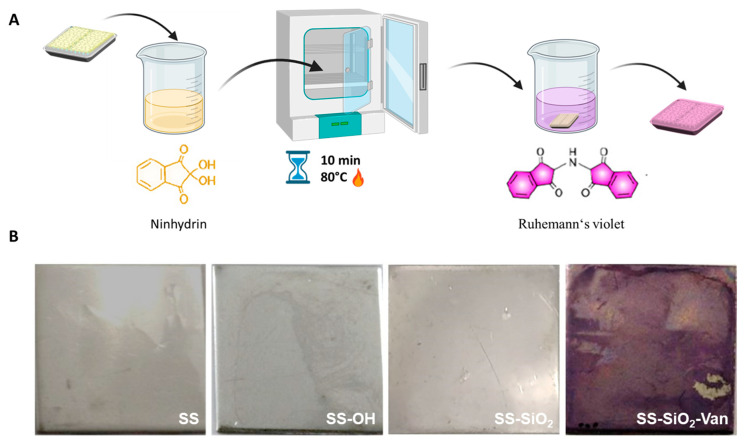
(**A**) Schematic illustration of ninhydrin colorimetric test; (**B**) Ninhydrin colorimetric assays for SS, SS-OH, SS-SiO_2_, and SS-SiO_2_-Van.

In addition, a colorimetric test was performed to assess the functionalization of the SS-SiO_2_-Van surface with the vanillin derivative **4**. For this purpose, ninhydrin was employed as a colorimetric testing agent. Ninhydrin is used for colorimetric detection, as it suffers a color change from yellow to deep purple in the presence of amine groups [58]. The deep-purple color, known as Ruhemann’s violet, is produced when two molecules of ninhydrin (2,2-dihydroxyindane-1,3-dione) react with a primary or secondary amine. Although the mechanism of this reaction is not entirely understood [59], it has been proposed that, in this reaction, the amine group undergoes a chemical reaction with ninhydrin, which acts as an oxidizing agent by initially causing oxidative deamination at high temperatures. Subsequently, a series of reactions between the products of the first reaction and new ninhydrin molecules lead to the formation of the deep-purple dikethydrin complex (Ruhemann complex) (Figure 4A) [58,59,60]. Considering this, the ninhydrin assay was performed by immersing the SS, SS-OH, SS-SiO_2_, and SS-SiO_2_-Van plates in the ninhydrin solution for 10 min at 80 °C. After this time, it was observed that only the SS-SiO_2_-Van plates turned purple (Figure 4B), confirming vanillin functionalization on the surface.

The quantification of functionalized vanillin on the surface of the SS-SiO_2_-Van was carried out by separating the colored Van–ninhydrin complex from the surface and its subsequent quantification using the adequate ninhydrin calibration curve, as explained in the experimental section. Following this approach, a functionalization of 0.95 ± 0.11 mg of vanillin per cm^2^ in SS-SiO_2_-Van was calculated. As expected, a 0 mg/cm^2^ value was found for SS, SS-OH, and SS-SiO_2_.

### 2.2. Antimicrobial Activity

**Figure 5:**
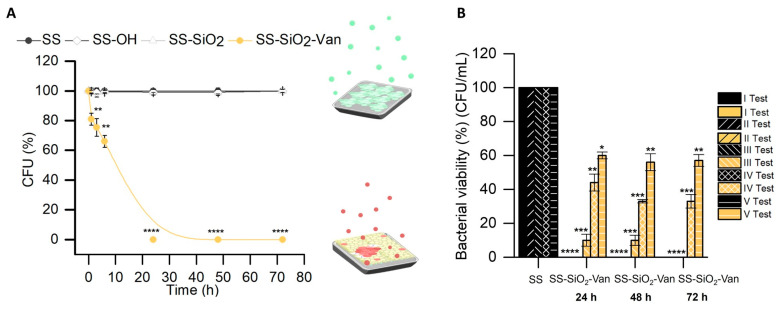
(**A**) *S. epidermidis* relative viability (% CFU) after contact with functionalized (SS-SiO_2_-Van) and non-functionalized (SS, SS-OH, and SS-SiO_2_) surfaces. Mean and standard deviation, n = 3. (**B**) Antimicrobial effect of reused SS-SiO_2_-Van surfaces against *S. epidermidis.* Statistical significance was determined by Student’s *t*-test for paired samples (* *p* < 0.05; ** *p* < 0.01; *** *p* < 0.001; **** *p* < 0.0001); n = 3.

The bacterial viability assays were carried out, as explained in the experimental section, by inoculating the functionalized surface material SS-SiO_2_-Van and the non-functionalized ones, SS, SS-OH, and SS-SiO_2_, with a suspension of *S. epidermidis*, subsequent incubation, and final counting of the number of colonies formed. The results obtained are shown in Figure 5A. The SS-SiO_2_-Van surfaces showed the most remarkable antimicrobial effect against *S. epidermidis*, leading to the total elimination of the microorganism after 24 h of treatment compared to untreated surfaces (SS, SS-OH, and SS-SiO_2_). After use, the surfaces were meticulously washed with ethanol and water, and analyzed with HR-FESEM. The EDX values obtained confirmed that the SS-SiO_2_-Van material still showed the presence of Si and N elements related to the presence of derivative **4**. The maintenance of the vanillin derivative in the SS surface after the antimicrobial assay confirms the covalent attachment of the natural antimicrobial to the metallic surface and, therefore, the mechanism of action of the developed antimicrobial surface based on the contact-killing approach.

After the successful antimicrobial assay with the SS-SiO_2_-Van material, the sturdiness of its activity was tested with successive bacterial viability assays. The efficacy of the antimicrobial activity of the SS-SiO_2_-Van material (Figure 5B) was fully maintained in the two following tests (II Test and III Test in Figure 5B), for which a total reduction in bacterial viability after a 72 h treatment was obtained. However, a decrease in the antimicrobial activity of the SS-SiO_2_-Van material was found in the following test cycles. Bacterial viability was reduced to ca. 33% and 60% for the IV Test and V Test after 72 h of incubation. We believe that the lower antimicrobial efficacy observed in subsequent cycles compared to the first ones is due to the intensive washing performed on the SS-SiO_2_-Van plates after each test, and also to the use of ultrasonication. These cleaning operations should not occur in natural situations, and, hence, the more extended durability of the antimicrobial activity is expected.

**Figure 6:**
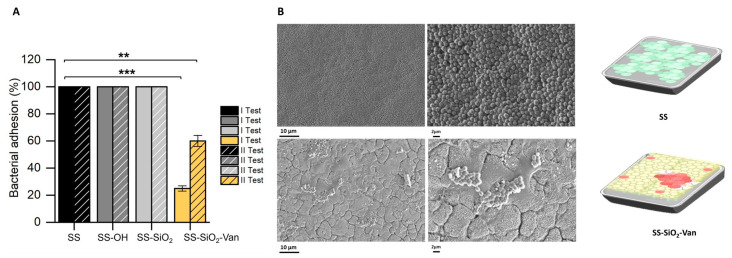
(**A**) I Test: Antimicrobial test to inhibit the bacterial adhesion of functionalized (SS-SiO_2_-Van) and non-functionalized (SS, SS-OH, and SS-SiO_2_) surfaces against *S. epidermidis* after 24 h of treatment. II Test: Antimicrobial test to inhibit the bacterial adhesion of reused SS-SiO_2_-Van surfaces against *S. epidermidis* after 24 h of treatment. Statistical significance was determined by Student’s *t*-test for paired samples (** *p* < 0.01; *** *p* < 0.001); n = 3. (**B**) HR-FESEM images of *S. epidermidis* adhesion after 24 h of incubation: untreated surface (SS) and modified surface (SS-SiO_2_-Van).

We have also evaluated the ability of the SS-SiO_2_-Van surfaces to inhibit bacterial adhesion and, therefore, to reduce the potential formation of biofilms. Bacterial growth and adhesion assays on the surfaces were performed as described in Section 3.6.2. As mentioned before, SS, SS-OH, and SS-SiO_2_ were used as negative controls. Once more, the study was conducted in a liquid medium to allow for contact between the bacteria and the functionalized or unfunctionalized surfaces during incubation; however, in this case, a culture medium (TSB) with the necessary nutrients to induce bacteria growth and the formation of biofilms was used. Once the SS, SS-OH, SS-SiO_2_, and SS-SiO_2_-Van plates were incubated with *S. epidermidis* for 24 h, the plates were gently washed to allow for the elimination of the bacteria that did not adhere. Then, plating was performed to enumerate the CFUs of the adhered bacteria on the surfaces. The results in Figure 6A (I Test) show that the SS-SiO_2_-Van surfaces were able to partially inhibit bacterial adhesion (the first step of biofilm formation) compared with the controls (SS, SS-OH, and SS-SiO_2_ plates).

Furthermore, the inhibition of the capacity of bacterial adhesion was additionally confirmed by the HR-FESEM images (Figure 6B). The comparison of the SS and SS-SiO_2_-Van surfaces showed a clear reduction in biomass for the latter after 24 h of contact with the microorganism *S. epidermidis*.

As in the previous experiment, the plates were washed thoroughly with ethanol and water, and analyzed with HR-FESEM-EDX. Here, again, the presence of the derivative **4** on the SS-SiO_2_-Van surface was confirmed, with the percentage content on Si and N found by the EDX analysis. To determine the maintenance of the inhibitory capacity against the growth of biofilms, the plates were reused for additional anti-adhesion experiments, and a ca. 40% inhibition of biofilm formation was obtained (Figure 6A, II Test). Although a lower anti-adhesion efficacy was observed in the second test, we believe this is due to the intensive washing performed on the SS-SiO_2_-Van plates after each test, which should not occur in natural situations.

**Figure 7:**
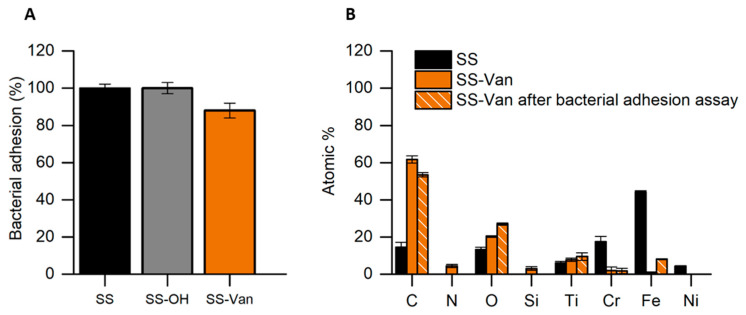
(**A**) Antimicrobial assay to inhibit bacterial adhesion of functionalized SS-Van (orange) and non-functionalized (SS and SS-OH) surfaces against *S. epidermidis* after 24 h of treatment. (**B**) HR-FESEM EDX analysis of SS-Van before (orange) and after (orange striped) bacterial adhesion assay.

To validate our functionalization process compared to the simple functionalization of the Van derivative on the SS surface, plates with a direct functionalization of the vanillin derivative (SS-Van plates) without the intermediate silica layer were also tested. As can be seen from the results shown in Figure 7, the antimicrobial activity is significantly lower for the SS-Van plates than for the plates with the intermediate silica layer SS-SiO_2_-Van (see Figure 6); in fact, only a 12% inhibition of bacterial adhesion is observed when using the SS-Van plates. Moreover, an even more important finding is that the HR-FESEM-EDX analysis after the bacterial adhesion assays showed the absence of Si and N on the surface of the SS-Van plates, indicating the leaching of derivative **4** during the essay. All of this demonstrates that coating the stainless steel with silica prior to the functionalization of the active molecule allows for the better anchoring of the derivative and, hence, gives our surfaces greater durability. In the case of the SS-Van plates, the covalent anchoring of the active molecule to the plate surface is obtained using Fe-O-Si covalent bonds, and this type of bond is not very robust because the Fe-O bond is very accessible to hydrolysis. However, in the case of our new functionalization process, the intermediate silica layer is firmly bonded to the steel surface by many covalent bonds, and the final anchoring of the active molecule takes place through Si-O-Si bonds, which are much more stable.

**Figure 8:**
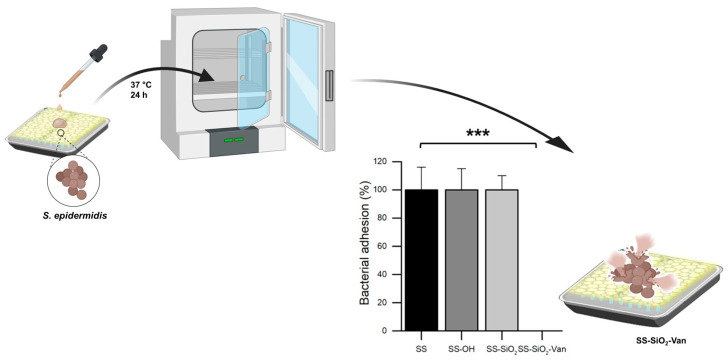
Antimicrobial test to inhibit the bacterial adhesion of functionalized (SS-SiO_2_-Van) and non-functionalized (SS, SS-OH, and SS-SiO_2_) surfaces against S. epidermidis after 24 h of treatment in semi-dry conditions. Statistical significance was determined by Student’s *t*-test for paired samples (*** *p* < 0.001); n = 3.

Finally, we also evaluated the ability of the SS-SiO_2_-Van surfaces to inhibit bacterial adhesion under semi-dry conditions. Exposure under semi-dry conditions was chosen to resemble typical exposure situations, such as oral/nasal splashes, contamination by droplets with a low organic content on surfaces in public spaces, non-invasive surfaces in healthcare environments, or work surfaces in the food industry. Bacterial growth and dry bacterial adhesion assays on the surfaces were performed as described in Section 3.6.3. As mentioned above, SS, SS-OH, and SS-SiO_2_ were used as negative controls. To emulate the semi-dry conditions of this study, a 20 µL drop of a previously prepared inoculum of *S. epidermidis* was deposited on the control and SS-SiO_2_-Van plates. Once the SS, SS-OH, SS-SiO_2_, and SS-SiO_2_-Van plates were incubated with *S. epidermidis* for 24 h, the plate was analyzed to enumerate the CFUs of the bacteria adhered to the surfaces. The results shown in Figure 8 show that, even under semi-dry conditions, the SS-SiO_2_-Van surfaces were able to 100% inhibit bacterial adhesion compared to the controls (SS, SS-OH, and SS-SiO_2_ plates). In fact, the results obtained in the adhesion test in semi-dry conditions were more effective than the adhesion test performed in a liquid medium. They are 1-fold more effective in semi-dry situations. We consider this test very important because it simulates what can occur in real situations in a hospital or food environment.

The in vitro results shown above may provide a potential solution for how to tackle the problem of the formation (on biotic and abiotic surfaces) of biofilms, by which bacteria protect themselves from external agents. We believe that this strategy can not only be used for *S. epidermidis* on stainless steel but can also be used on other different surfaces and for other bacteria, since vanillin, as widely described in the literature, is known for its antimicrobial activity against many types of microorganisms [27,37,61,62]. We believe that similar procedures can be used for the design of antimicrobial prostheses or medical devices, and for applications to prevent microbial contaminations in the food industry. The use of natural molecules can also minimize the risks of traditional sanitizers and synthetic antimicrobials. As we can observe in Table 1, many studies have dealt with the functionalization of surfaces with natural products. However, as we can see, our surfaces not only have better antimicrobial efficacy, reaching 100% inhibition within 24 h of contact, but they also have greater durability over time. This durability, as we have shown above, is due to the coating of the surface with an intermediate silica layer, which allows for a more stable anchoring of the antimicrobial molecule. Our studies demonstrate that EOCs can be used to design highly effective antimicrobial coatings.

## 3. Materials and Methods

### 3.1. Reagents, Bacterial Strain, and Culture Media

Tetraethylorthosilicate (TEOS), sodium hydroxide (NaOH), (3-aminopropyl) triethoxysilane (APTES), vanillin (Van), and palladium on carbon (Pd/C) were provided by Sigma (Sigma-Aldrich Química S.L., Madrid, Spain). Ethanol (extra-pure), dichloromethane (DCM), acetone, hydrogen peroxide, and sulfuric acid were purchased from Scharlab S.A. (Barcelona, Spain). Ninhydrin was provided by PanReac Quimica SA (Barcelona, Spain). AISI 316L stainless steel plates (20 mm × 20 mm × 2 mm) were purchased from Gimetal (Valencia, Spain).

*S. epidermidis* (RP62A) was obtained from the Spanish Type Culture Collection (CECT, Burjassot, Valencia, Spain). The bacterial strain was reconstituted according to the CECT’s instructions for bacterial viability and adhesion assays. The CECT protocol is the same as that used by the ATCC (American Type Culture Collection). After that, the bacterial stock was maintained at 4 °C in plate count agar before its use. *S. epidermidis* inoculum was prepared by placing one single colony of the *S. epidermidis* strain into 10 mL of tryptic soy broth (TSB). The mixture was incubated at 37 °C for 24 h to obtain an inoculum with a density of approximately 10^8^ CFU/mL of broth. All of the culture media were supplied by Scharlab S.A. (Barcelona, Spain).

### 3.2. Synthesis of the Vanillin Derivate

An amount of 1 g (6.57 mmol) of vanillin (**1**) was suspended in 25 mL of dichloromethane, and then 1.2 mL (5.13 mmol) of APTES (**2**) was added to the mixture. The mixture was stirred for 2 h, and, after this, the suspension was evaporated under reduced pressure to obtain the corresponding alkoxysilane derivative, (E)-2-methoxy-4-(((3-(triethoxysilyl)propyl)imino)methyl)phenol, derivative **3** (Figure 3B).

^1^H NMR (400 MHz, DMSO) δ: 8.12 (s, 1H), 7.29 (d, J = 1.8 Hz, 1H), 7.07 (dd, J = 8.1, 1.8 Hz, 1H), 6.78 (d, J = 8.1 Hz, 1H), 3.76 (d, J = 2.2 Hz, 3H), 3.74–3.68 (m, 6H), 3.44 (dt, J = 11.3, 6.8 Hz, 2H), 1.66–1.58 (m, 2H), 1.11 (dd, J = 9.0, 5.0 Hz, 9H), and 0.57–0.51 (m, 2H).

^13^C NMR (400 MHz, DMSO) δ: 160.54, 149.89, 128.14, 123.05, 115.62, 110.24, 58.05, 56.49, 55.35, 24.59, 18.49, and 9.1 ppm.

HRMS, calculated for C_17_H_29_NO_5_Si: 355.1815, found: 356.1888 (M + H^+^).

In a second step, the alkoxysilane derivate **3** (5 mmol) was dissolved in ethanol, and 340 mg of Pd/C was added. Subsequently, N_2_ gas flow was passed through the reaction flask to displace the O_2_. Then, H_2_ flow was added. The mixture was stirred for 3 h under an inert atmosphere. Finally, the reduced derivate, 2-methoxy-4-(((3-(triethoxysilyl)propyl)amino)methyl)phenol (Van-APTES), derivative **4** (Figure 3B), was filtered off and evaporated under reduced pressure to obtain the pure compound as a pale-yellow solid in a 99% yield.

^1^H NMR (400 MHz, DMSO) δ: 6.88 (s, 1H), 6.70–6.63 (m, 2H), 3.75–3.72 (m, 6H), 3.71 (s, 3H), 3.45 (d, J = 7.0 Hz, 2H), 2.45 (t, J = 7.1 Hz, 2H), 1.52–1.39 (m, 2H), 1.17–1.10 (m, 9H), and 0.60–0.51 (m, 2H).

^13^C NMR (400 MHz, DMSO) δ: 147.81, 145.62, 131.98, 121.39, 115.7, 113.58, 57.9, 53.10, 51.70, 23.15, 18.50, and 8.03 ppm.

^29^Si NMR (400 MHz, DMSO) δ: −44.90 ppm.

HRMS, calculated for C_17_H_31_NO_5_Si: 357.1971, found: 358.2044 (M + H^+^).

### 3.3. Synthetic Immobilization of the EOC on the Stainless Steel Surface

#### 3.3.1. Pretreatment of Stainless Steel Surface

Stainless steel plates (SS plates) were placed in a beaker (1 plate in 1 beaker) and sonicated for 10 min in a mixture of acetone/water (70% *v*/*v*), ethanol, and then deionized water. After sonication, a treatment with piranha solution was performed to activate the SS plates. To prepare the corresponding piranha solution, 30% of hydrogen peroxide was poured into a beaker, and concentrated sulfuric acid was slowly added to finally obtain a 7:3 (*v*/*v*) sulfuric acid to hydrogen peroxide ratio mixture. The cleaned SS plates were immersed in the piranha solution for 10 min. After this, the plates were removed and placed in a second piranha solution for 5 min. After the second piranha treatment, the SS plates were rinsed twice in ultrapure water before being placed in an ultrapure water bath for 24 h. The resulting plates are referred to as SS-OH.

#### 3.3.2. Deposition of the Silica Layer

The SS-OH plates were placed in a basic aqueous solution and heated at 80 °C. Then, 5 mL of TEOS were added drop by drop, and the mixture was stirred for thirty minutes. The plates were rinsed twice in ultrapure water and dried at 100 °C overnight. The resulting plates are referred to as SS-SiO_2_.

#### 3.3.3. Immobilization of the Vanillin Derivative

An amount of 1 g of Van-APTES (vanillin derivative) was solubilized in ethanol. The solution was poured onto the SS-SiO_2_ plates and shaken for 5.5 h at room temperature. Then, the plates were washed with ethanol and dried at room temperature. The resulting plates are referred to as SS-SiO_2_-Van.

#### 3.3.4. Immobilization of the Vanillin Derivative Directly on the SS (Without the Silica Layer)

The surfaces were activated as in Section 3.3.1. An amount of 1 g of Van-APTES was solubilized in ethanol. The solution was poured onto the SS-OH plates and stirred for 5.5 h at room temperature. The plates were then washed with ethanol and dried at room temperature. The resulting plates are referred to as SS-Van.

### 3.4. Characterization Methods

Functionalized surfaces and trialkoxysilane derivatives were characterized using standard techniques. Trialkoxysilane derivatives were characterized by the Bruker Fourier Transform Nuclear Magnetic Resonance spectroscopy (FT-NMR) Advance 400 (Ettlingen, Germany) at 300 K and analyzed with MestReNova 6.0 software. A quartz tube was used for ^29^Si to reduce possible interference with the Si present in the glass tubes generally used for NMR analysis. The Si signal of the quartz tubes is much narrower than that of the glass tubes and can be differentiated from the signal of our product. Electron impact ionization mass spectrometry (MS-EI) was performed on a Thermo Finnigan MAT SSQ710 (Thermo Fisher Scientific, Rafelbunyol, Valencia, Spain) single-stage quadrupole instrument, and high-resolution mass spectra (HRMS) were carried out in a TRIPLETOF T5600 (AB Sciex, Framingham, Massachusetts, USA) spectrometer. The composition of the surface-functionalized substrates was determined by a High-Resolution Field Emission Scanning Electron Microscope (HR-FESEM). The HR-FESEM images and Energy-Dispersive X-ray Spectrometry (EDX) analysis were obtained with a GeminiSEM 500 microscope (ZEISS OXFORD INSTRUMENTS, Oxford, England). Imaging and analysis were acquired with an In-lens detector (1.5 kV, a 5 mm working distance, and a 30 µm aperture size (standard aperture)). Ultraviolet–visible (UV-Vis) spectroscopy was used to quantify the functionalization of the surfaces. UV-Vis spectroscopy measurements were performed with a JASCO V-630 Spectrophotometer (JASCO, Easton, PA, USA).

### 3.5. Quantification of the Functionalized EOC

The SS-SiO_2_-Van plates were immersed in a ninhydrin solution (8 mg of ninhydrin dissolved in 10 mL of ethanol) for 10 min at 80 °C. After that, the SS-SiO_2_-Van plates were immersed in 5 mL of ethanol and sonicated for several hours to separate the Van–ninhydrin complex from the surface. When all of the color on the SS plates due to the attached ninhydrin was released, the Van–ninhydrin content of the ethanol solvent was quantified by measuring its absorbance at 567 nm. A calibration curve with the Van–ninhydrin complex obtained by reacting the ninhydrin with the Van-APTES derivative in ethanol (from 0.9 to 10.5 mg/mL) had been previously prepared and was used for quantification. The same methodology was performed for the bare and control surfaces: SS plates (bare surface), SS-OH plates, and SS-SiO_2_ (control surfaces). All of the tests were carried out in triplicate.

### 3.6. Microbiological Analysis

Two tests were performed to assess the effect of surface functionalization, first against bacterial cell growth (viability assays) and, in second place, against bacterial cell adhesion (bacterial adhesion assays for biofilm). Antimicrobial tests for the modified surfaces were performed against *S. epidermidis*.

#### 3.6.1. Bacterial Viability Assays

The antimicrobial efficiency was performed in the liquid medium PBS. Inoculum dilutions of *S. epidermidis* were prepared in PBS to obtain a concentration of 10^4^ cells/mL. A flask containing the functionalized SS-SiO_2_-Van surfaces was inoculated with 1 mL of *S. epidermidis* suspension to obtain a final concentration of 10^3^ cells/mL. The SS, SS-OH, and SS-SiO_2_ plates were treated following the same procedure as for SS-SiO_2_-Van. Finally, all of the samples were incubated with orbital shaking (120 rpm) at 37 °C for 24 h. To enumerate viable cells, 100 µL aliquots were taken from each suspension (SS, SS-OH, SS-SiO_2_, and SS-SiO_2_-Van) and distributed on Tryptone plates (TSA). After plate incubation at 37 °C for 24 h, the colonies were counted, and the obtained results were expressed as a percentage of CFUs (see Tables S1 and S2 for the CFU raw data). All of the treatments were tested in triplicate.

#### 3.6.2. Bacterial Adhesion Assays

Bacterial adhesion assays were performed in a Tryptic Soy Broth (TSB) liquid medium. Inoculum dilutions of *S. epidermidis* were prepared in TSB to obtain a concentration of 10^4^ cells/mL. A flask containing the functionalized SS-SiO_2_-Van plates was inoculated with 1 mL of *S. epidermidis* suspension to obtain a final concentration of 10^3^ cells/mL. SS, SS-OH, SS-SiO_2_, and SS-Van plates were also immersed in a bacterial suspension similar to that of the control. Finally, all of the samples were incubated at 37 °C for 24 h. After incubation, unattached bacteria were removed by gently washing the plates with sterile PBS (pH of 7.4). Each plate with adherent bacteria was placed in a flask containing 3 mL of sterile PBS and sonicated for 5 min. The sonicated solutions containing the recovered bacteria were serially diluted, and 100 µL of each dilution was distributed onto Tryptic Soy Agar (TSA) plates. The plates were incubated overnight at 37 °C, and the number of colonies was counted. The bacteria viability was expressed as % of CFUs (see Table S3 for the CFU raw data). All of the treatments were tested in triplicate.

#### 3.6.3. Dry Bacterial Adhesion Assays

Bacterial adhesion assays were also performed in dry conditions. Inoculum dilutions of *S. epidermidis* were prepared in TSB to obtain a concentration of 10^4^ cells/mL. Functionalized SS-SiO_2_-Van plates were placed in a 6-well plate and inoculated with 20 µL droplets of *S. epidermidis*. SS, SS-OH, and SS-SiO_2_ plates were used as control plates and consequently treated similarly. Finally, all of the samples were incubated at 37 °C for 24 h. After incubation, the bacteria were placed in a flask containing 3 mL of sterile PBS and sonicated for 5 min. The sonicated solutions containing the recovered bacteria were serially diluted, and 100 µL of each dilution was spread onto TSA plates. The plates were incubated overnight at 37 °C, and the number of colonies was counted. The viability of the bacteria was expressed as % CFUs (see Table S4 for the CFU raw data). All of the treatments were tested in triplicate.

### 3.7. Statistical Analysis

The statistical analysis of the results was performed with Statgraphics Centurion XVIII (Statpoint Technologies, Inc., Warrenton, VA, USA). The differences observed between the controls (SS, SS-OH, and SS-SiO_2_ plates) and SS-SiO_2_-Van surfaces with regard to the microbial viability and bacterial adhesion assays were analyzed using Student’s *t*-test for paired samples. Significant statistical differences were indicated by the corresponding asterisks (* *p* < 0.05, ** *p* < 0.01, *** *p* < 0.001, and **** *p* < 0.0001).

## 4. Conclusions

In summary, we present here an innovative antimicrobial treatment for stainless steel surfaces, in which the surfaces were first coated with a silica layer and then functionalized with vanillin. The antimicrobial and antibiofilm surface obtained is environmentally and human-health-friendly, as vanillin is a natural compound registered under the REACH-EACH (Registration, Evaluation, Authorization and Restriction of Chemicals at the European Chemical Agency) Regulation. Specifically, we focused our study on the efficacy of the vanillin-functionalized surfaces against the Gram-positive bacterium *S. epidermidis*, one of the most frequent causes of nosocomial infections. We have demonstrated a high antimicrobial effect of the developed coating against *S. epidermidis* when it comes into contact with the vanillin-functionalized stainless steel surfaces, recording a 100% reduced bacterial viability. Moreover, we have also shown that surfaces functionalized with vanillin and pre-coated with a silica layer showed a much greater ability to inhibit bacterial adhesion (the first step of biofilm formation) not only compared to untreated surfaces but also compared to surfaces directly functionalized with Van-APTES (SS-Van). SS-Van surfaces were shown to have poor antimicrobial activity and especially poor durability. This could be understood because, in the case of SS-Van, the covalent bond that anchors the antimicrobial molecule is of the type Fe-O-Si, which is not very robust against hydrolysis. In the case of our double-coating procedure, the silica layer strengthens the anchorage of Van to the steel, being the final anchorage through Si-O-Si bonds, which are much more stable. Furthermore, we have demonstrated that bacterial adhesion under semi-dry conditions, which simulate possible real conditions, is 100% inhibited, confirming the high efficacy of our functionalized surfaces. Finally, we would like to stress that our approach is highly versatile due to, on the one side, the broad spectrum of the vanillin antimicrobial properties and, on the other side, the easy application of the double-coating functionalization process to other EOCs or natural molecules of interest. We believe that EOC-functionalized stainless steel surfaces can contribute to designing new strategies to address nosocomial infections and the formation of resistant, difficult-to-eliminate structures such as microbial biofilms on surfaces. Potential applications in the medical field and food industry are envisioned. We hope this research will open the door to future studies on new functionalization with EOCs on surfaces and their use in numerous applications.

## Figures and Tables

**Table 1:** Antimicrobial effect of surfaces functionalized with essential oil components (EOCs).

Surface	Natural Antimicrobial	Microorganisms	Results	Durability	References
Plastic flexible films	Microcapsules containing carvacrol and thymol	*E. coli*, *S. aureus*, *L. innocua*, *S. cerevisiae*, and *A. niger*	Thymol and carvacrol showed significant antimicrobial activity against the studied microorganisms, with minimal inhibitory concentrations (MICs) of 125–250 ppm and 75–375 ppm for thymol and carvacrol, respectively. The concentration of the microencapsulated antimicrobial agents showed a range of inhibition zones of 4.3–11.3 mm for the microorganisms at 10% thymol and 10% carvacrol.	28 days	[39]
Chitosan	Cinnamaldehyde	*S. aureus* and *E. coli*	The films’ effectiveness increased as the treatment temperature increased, and, thus, the amount of cinnamaldehyde was released. Treatment at 4 °C for 30 min showed reduced antimicrobial activity (1 log reduction). After treatment at 65 °C for 30 min, the films showed a significant log reduction of 5.66 ± 0.04 against *S. aureus* and 4.76 ± 0.02 against *E. coli*. It was also observed that the films treated at 72 °C for 15 min, 95 °C for 10 min, and 121 °C for 5 min produced a bactericidal effect.	Tested 24 h	[41]
Stainless steel and titanium	Mentha piperita	*E. coli*	The mint coating was able to reduce to 1–2 Log of CFUs after 24–48 h, demonstrating a bacteriostatic effect.	Tested 48 h	[42]
Titanium	Peppermint oil	*Staphylococci*	Bacteriostatic effect (able to reduce approximately 1–2 Log of CFUs after 24 h).	Tested 24 h	[43]
Stainless steel	Carvacrol and eugenol	*P. aeruginosa* and *C. albicans*	The carvacrol coating has a biofilm growth reduction rate of up to 44% for *P. aeruginosa* and 60% for *C. albicans*. Similarly, the eugenol coating exhibited up to 36% suppression for *P. aeruginosa* and 52% for *C. albicans*. Reduced in both cases approximately 1–2 log of CFUs.	Tested 24 h	[44]

## Data Availability

Data will be made available on request to the authors.

## Supplementary Materials

The following supporting information can be downloaded at: https://www.mdpi.com/article/10.3390/ijms252212146/s1.
