# Supplementary material for: Antimicrobial Surfaces: Stainless Steel Functionalized with the Essential Oil Component Vanillin
Source: Int J Mol Sci. 2024 Nov 12;25(22):12146. doi: 10.3390/ijms252212146 (PMC11595243; doi:10.3390/ijms252212146)
Supplement: Supplementary file 1 [file ijms-25-12146-s001.zip › ijms-3111979-supplementary.pdf]

Antimicrobial Surfaces: Stainless Steel Functionalized with the Essential Oil Component Vanillin

Serena Medaglia [1,2], Ángela Morellá-Aucejo [1,2,3], María Ruiz-Rico [4], Félix Sancenón [1,2,3,5,6], Luis A. Villaescusa [1,2,5], Ramón Martínez-Máñez [1,2,3,5,6], M. Dolores Marcos [1,2,3,5,6,*] and Andrea Bernardos [1,2,3,5,*]

1. Microbiological analysis

Table S1. Data CFU of *S. epidermidis* viability after contact with functionalized (SS-SiO_2-Van) and non-functionalized (SS, SS-OH and SS-SiO_2) surfaces.

	CFU (1 h)	CFU (3 h)	CFU (6 h)	CFU (24 h)	CFU (48 h)	CFU (72 h)
SS	3.85	3.90	3.89	4.0	4.0	4.90
SS-OH	3.90	3.90	3.99	3.99	4.22	4.88
SS-SiO_2	3.89	3.90	3.90	4	4.20	4.90
SS-SiO_2-Van	3.19	2.66	2.34	0	0	0

Table S2. Data CFU of antimicrobial effect of reused SS-SiO_2-Van surfaces against *S. epidermidis.*

	Controls	SS-SiO_2-Van	Controls	SS-SiO_2-Van	Controls	SS-SiO_2-Van
	CFU (24h)		CFU (48 h)		CFU (72 h)	
I Test	3.85	0	4.10	0	4.60	0
II Test	3.88	0	3.99	0	4.45	0
III Test	3.80	0.5	4.0	0	4.63	0
IV Test	3.84	1.7	4.0	1.3	4.50	1.60
V Test	3.86	2.3	3.99	1.9	4.50	2.22

Table S3. Data CFU of antimicrobial test to inhibit bacterial adhesion of functionalized (SS-SiO_2-Van) and non-functionalized (Controls) surfaces against *S. epidermidis* after 24 h of treatment (I test and II Test).

	I Test (CFU)	II Test (CFU)
Controls	8.30	8.22
SS-SiO_2-Van	2.12	5.10

Table S4. Data CFU of antimicrobial test to inhibit bacterial adhesion of functionalized (SS-SiO_2-Van) and non-functionalized (SS, SS-OH and SS-SiO_2) surfaces against *S. epidermidis* after 24 h of treatment in semi-dry conditions.

	SS	SS-OH	SS-SiO_2	SS-SiO_2-Van
CFU (24 h)	3.89	3.88	4.00	0
